# Supplementary material for: Terrain Ruggedness and Canopy Height Predict Short-Range Dispersal in the Critically Endangered Black-and-White Ruffed Lemur
Source: Genes (Basel). 2023 Mar 18;14(3):746. doi: 10.3390/genes14030746 (PMC10048730; doi:10.3390/genes14030746)
Supplement: Supplementary file 1 [file genes-14-00746-s001.zip › genes-2168746-supplementary.pdf]

Supplemental Materials, Figures, and Tables for:

Terrain ruggedness and canopy height predict short-range dispersal in the Critically Endangered black-and-white ruffed lemur

Amanda N. Mancini[1,2], Aparna Chandrashekar[1,2], Andrea L. Baden[1,2,3]

[1] Department of Anthropology, The Graduate Center, City University of New York, 365 Fifth Ave, New York, NY 10016 USA

[2] The New York Consortium in Evolutionary Primatology (NYCEP)

[3] Department of Anthropology, Hunter College, 695 Park Avenue, New York, NY 10065 USA

SUPPLEMENTAL METHODS S1

Forest Cover Classifications

To generate classifications of 1990 (historic) and 2016 (contemporary) forest cover throughout the Ranomafana National Park and COFAV region we acquired relatively cloud-free Landsat scenes from the Landsat TM (Thematic Mapper) and OLI (Operational Land Imager) sensors at four time periods: 1989-90 (TM; henceforth 1990), 1997-98 (TM; henceforth 1998), 2008-09 (TM: henceforth 2009), and 2015-16 (OLI; henceforth 1990). Two scenes at each time point were acquired during the dry season from late October to early November to maintain a similar solar azimuth angle between scenes and cloud coverage in all scenes was less than 10% (Table SVI). All Landsat scenes were converted to top-of-atmosphere (TOA) reflectance and scenes from each period were mosaicked together using a feathering distance of 50 pixel, no color correction or histogram matching, no seamlines generated, and data values of 0 were ignored; TOA conversion and scene mosaics were conducted in ENVI 55. To co-register the scenes, mosaicked images from all time periods were layer-stacked and cropped to the approximate extend of the study area in ENVI Classic 55.

Spectral mixture analysis (SMA) and linear spectral unmixing were used to convert mosaicked scenes from each period into scenes representing the abundance of three landcover types: high albedo surfaces (e.g., substrate), vegetation, and dark albedo surfaces (e.g., water or shadow). Almost all pixels in an image are mixed pixel (i.e., mixed radiance from multiple landcover types), and SMA used a linear mixture model to simulate the physical process of a mixed radiance measurement in the instantaneous field of view (IFOV) of a sensor. The linear model represents the reflectance factor (R) of each pixel as a linear combination of the abundances (or fractions; f) of each landcover type

(endmember) multiplied by the endmember spectral profile (M) plus a misfit (ε) as:

$$R = fM + \varepsilon \qquad \text{(eq. 1)}$$

By inverting the linear model with linear spectral unmixing we can quantify the fractions (f) of each landcover type to generate a physical representation of landcover across the study extent [135,136]. Using global endmembers from Small (2004) [136] of high albedo, vegetation, and dark albedo surfaces we unmixed mosaicked images from all four time periods. Endmembers from Small (2004) [136] were derived from a global composite of 30 spectrally diverse subscenes and were used to enable greater generality of analyses, rather than deriving scene-specific endmembers.

To generate thematic classifications of landcover, we used a custom-built decision tree algorithm in ENVI Classic 55 to partition spectrally unmixed pixels into six categories (Figure S7; Friedl & Brodley, 1997): shadow, water, cloud, substrate, non-forest vegetation, and forest. Decision boundaries were generated through visual inspection of the unmixed feature space comparing each combination of the three endmember fractions (Figures S6 and S7) [133]. Boundaries between substrate, non-forest vegetation, and forest were continuous, however forest was mainly composed of vegetation mixed with dark albedo (e.g., shadow) fractions, non-forest vegetation was mainly composed of vegetation mixed with high albedo fractions, and substrate was mainly composed of high albedo fractions. These physical mixtures of endmember fractions coincide with the physical expectations of each landcover type. Thematic classifications were all generated at the native 30 m resolution and exported from ENVI as a TIFF file for later processing.

Using QGIS v.3.20, thematic classifications were masked to the final study extent.

Cloud, shadow, and river classes in the 1990 and 1998 classifications were filled using the classification from the subsequent period (1998 and 2009, respectively). Using the raster calculator, pixels identified as clouds, shadows, or river were assigned the class value of the later time point (e.g., cloud in 1990 took the value of the corresponding pixel from 1998) and all other classes remained the same. Given that little forest regeneration occurs on the island [126,134], the fills were likely conservative in terms of estimating remaining forest cover which is the primary motivation in these classifications. Finally, thematic classifications were reclassified into binary forest-matrix classifications. The classifications from 1990 and 2016 were used to evaluate the role historic and contemporary forest cover on ruffed lemur dispersal below.

References

126. Vieilledent, G.; Grinand, C.; Rakotomalala, F.A.; Ranaivosoa, R.; Rakotoarijaona, J.R.; Allnutt, T.F.; Achard, F. Combining global tree cover loss data with historical national forest cover maps to look at six decades of deforestation and forest fragmentation in Madagascar. Biol. Conserv. 2018, 222, 189–197.
133. Friedl, M.A.; Brodley, C.E. Decision Tree Classification of Land Cover from Remotely Sensed Data. Remote Sensing of Envi-ronment. 1997, 61, 399–409.
134. Hansen, M.C., Potapov, P.V., Moore, R., Hancher, M., Turubanova, S.A., Tyukavina, A., Thau, D., Stehman, S.V., Goetz, S.J., Loveland, T.R.; et al. High-resolution global maps of 21st-Century forest cover change. Science. 2013, 342, 850–853.
135. Small, C. The Landsat ETM+ spectral mixing space. Remote Sensing of Environment. 2004, 93, 1–17.
136. Sousa, D.; Small, C. Global cross-calibration of Landsat spectral mixture models. Remote Sensing of Environment. 2017, 192, 139–149.

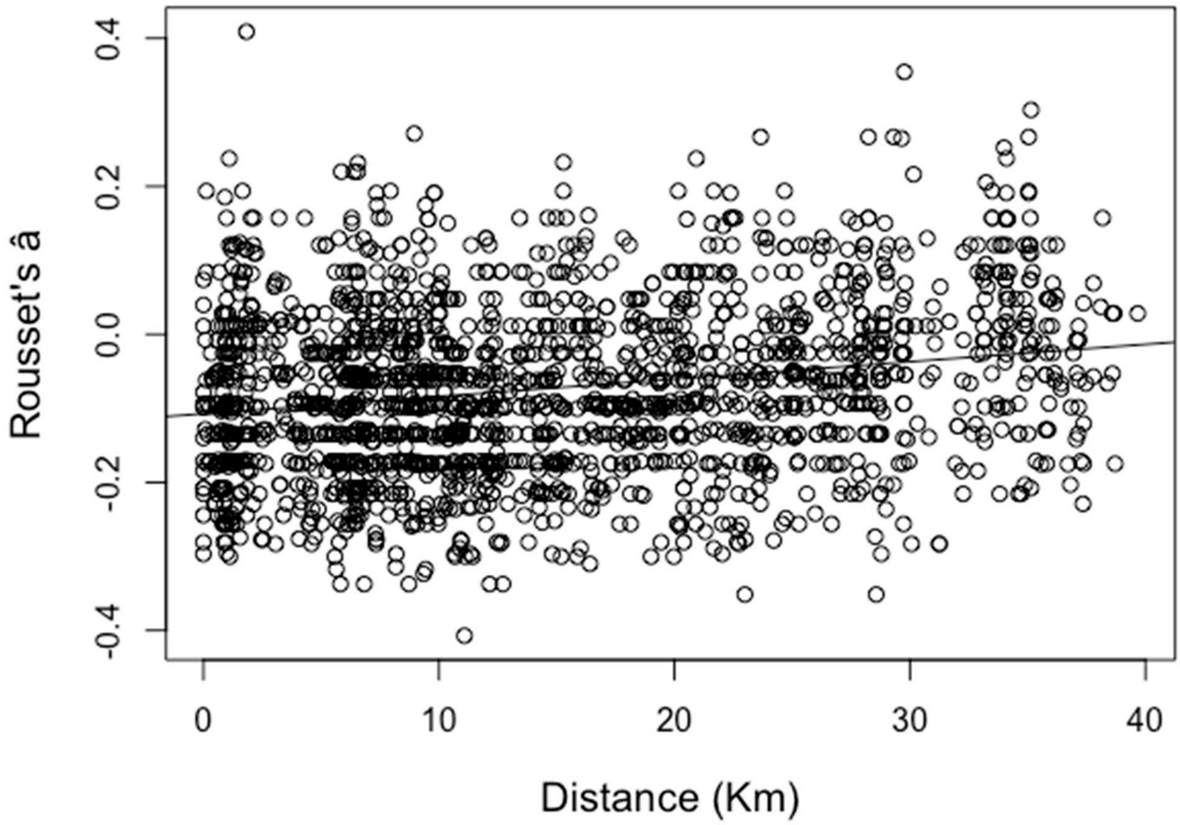

Figure S1. Linear regression between pairwise Euclidean and genetic (Ar) distances. Isolation-by-distance relationship was significant and explained 4.4% (R-value = 0.209; Multiple R^2 = 0.0437; mean 95% CI: 0.1655, 0.2517) of the observed population genetic structure.

Table S1. Parameter estimates from mixed effects model fit to the optimized singular resistance surfaces. All resistance values were scaled and centered by subtracting the mean and dividing by the standard deviation of resistance values. Rows shown in bold indicate models that performed better than Euclidean distance alone and were selected as the top model more than 1% ($\hat{\pi} \geq 1.00$) during 10,000 bootstrap iterations.

Resistance Surface	Parameter	β	S.E.	t-value
1990 Forest Cover	Intercept	-0.073	0.013	-5.565
	Forest	0.020	0.002	8.464
2016 Forest Cover	Intercept	-0.073	0.013	-5.546
	2016 Forest	0.020	0.002	8.467
Altitude	Intercept	-0.073	0.013	-5.561
	Altitude	0.020	0.002	8.452
**Canopy Cover**	**Intercept**	**-0.073**	**0.013**	**-5.653**
	**Canopy Cover**	**0.023**	**0.003**	**8.652**
Fire Density	Intercept	-0.073	0.013	-5.557
	Fire Density	0.019	0.003	8.419
Rivers	Intercept	-0.073	0.013	-5.547
	Rivers	0.020	0.002	8.438
**TPI†**	**Intercept**	**-0.073**	**0.013**	**-5.597**
	**TPI†**	**0.022**	**0.003**	**8.550**
**TRI‡**	**Intercept**	**-0.073**	**0.012**	**-5.825**
	**TRI‡**	**0.029**	**0.003**	**8.784**

†TPI: Topographic Position Index

‡TRI: Terrain Ruggedness Index

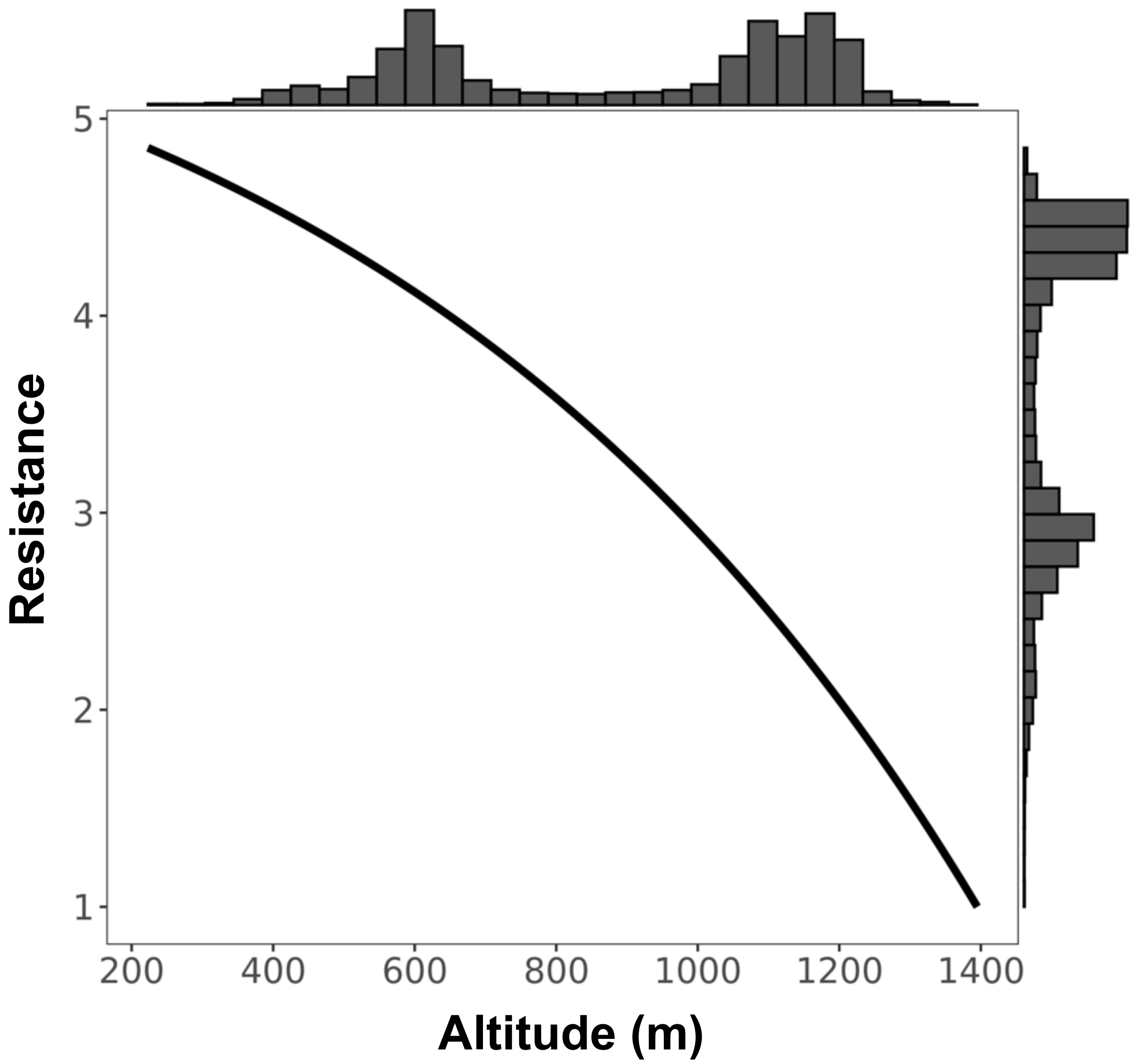

Figure S2. Resistance transformation plot for single surface altitude. Resistance to ruffed lemur movement deceased monotonically with increasing altitude.

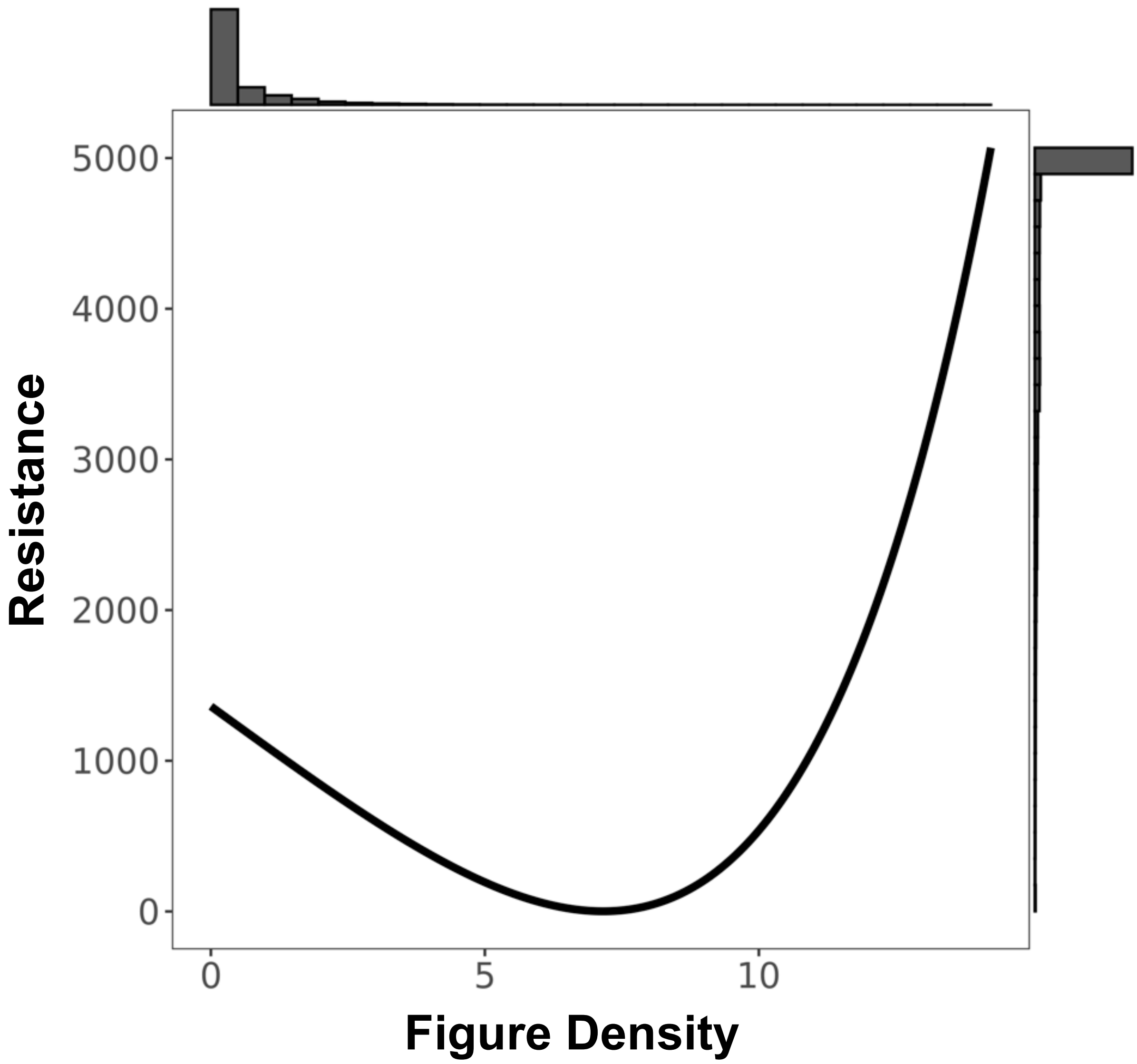

Figure S3. Resistance transformation plot for single surface fire density. Resistance to ruffed lemur movement increased exponentially with increasing fire density.

Table S2. Spearman rank correlation between singular resistance surfaces. Surfaces in bold are those that performed better than Euclidean distance alone and were selected as the top model more than 9% ($\hat{\pi} \geq 9.00$) during 10,000 bootstrap iterations. Correlation values range from ρ = 0.00 (uncorrelated) to ρ = 1.00 (monotonic correlation).

	**TRI†**	**Canopy Height**	TPI‡	1990 Forest Cover	2016 Forest Cover	Altitude	Rivers
**Canopy Height**	**0.078**	--	--	--	--	--	--
**TPI‡**	**0.082**	**0.071**	--	--	--	--	--
1990 Forest Cover	0.119	0.438	0.175	--	--	--	--
2016 Forest Cover	0.038	0.767	0.084	0.419	--	--	--
Altitude	-0.190	0.529	0.130	0.381	0.547	--	--
Rivers	-0.089	-0.130	-0.158	-0.142	-0.104	-0.177	--
Fire Density	0.049	-0.336	-0.011	-0.016	-0.330	-0.213	0.001

†TRI: Terrain Ruggedness Index

‡TPI: Topographic Position Index Ruggedness Index

Table S3. Parameter estimates from mixed effects model fit to the optimized composite resistance surfaces. All resistance values were scaled and centered by subtracting the mean and dividing by the standard deviation of resistance values. Rows shown in bold indicate models that performed better than Euclidean distance alone and were selected as the top model more than 10% ($\hat{\pi} \geq 10.00$) during 10,000 bootstrap iterations.

Resistance Surface	Parameter	β	S.E.	t-value
Combination A	Intercept	-0.073	0.013	-5.685
	Combination A	0.023	0.003	8.636
Combination B	Intercept	-0.073	0.013	-5.651
	Combination B	0.023	0.003	8.657
**Combination C**	**Intercept**	**-0.073**	**0.012**	**-5.816**
	**Combination C**	**0.030**	**0.003**	**8.788**
**Combination D**	**Intercept**	**-0.073**	**0.012**	**-5.812**
	**Combination D**	**0.030**	**0.003**	**8.782**

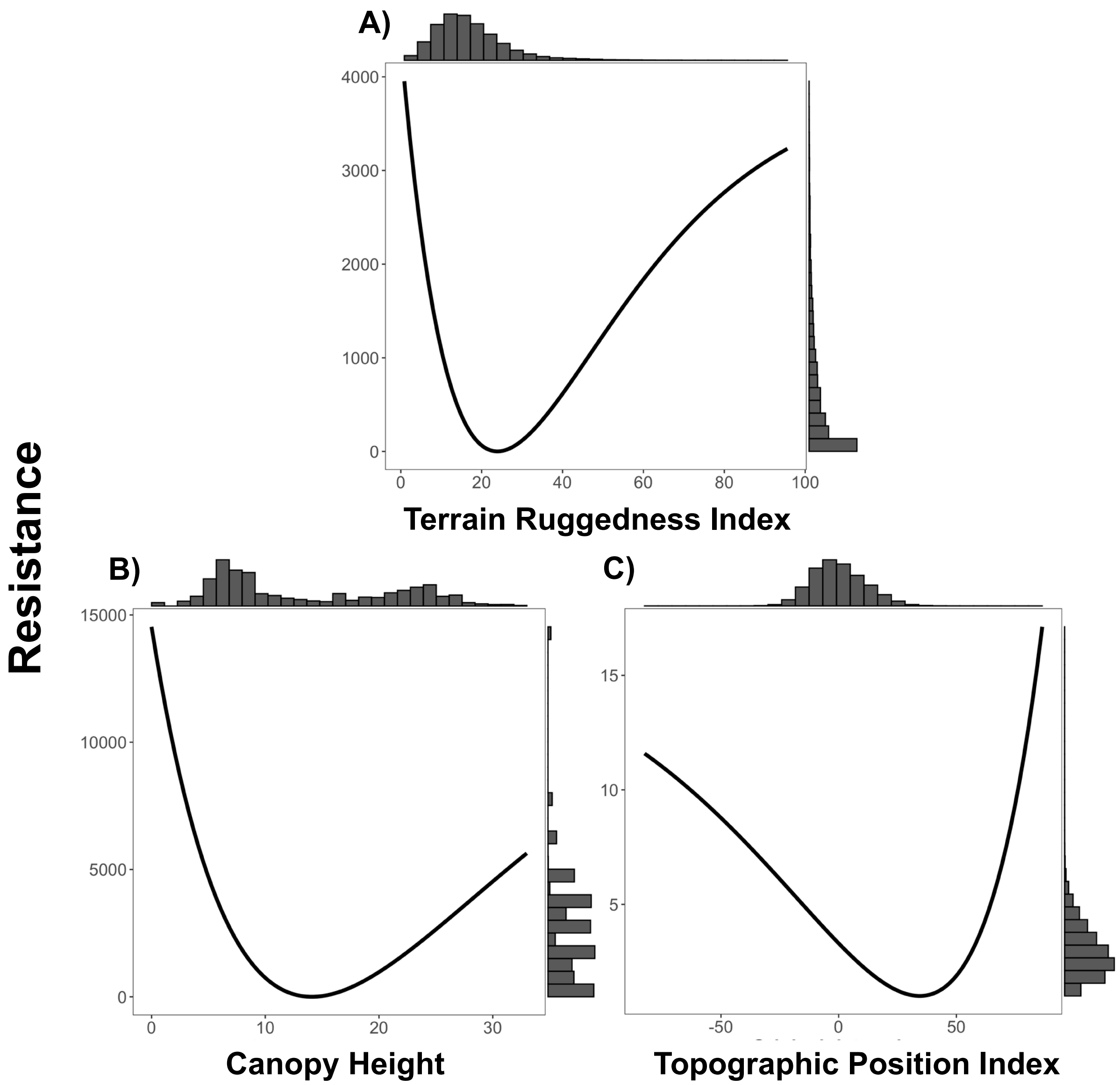

Figure S4. Resistance transformation plot for 'Composite A' surface of (A) Terrain Ruggedness Index (TRI), (B) canopy height, and (C) Topographic Position Index (TPI). Low TRI values represent relatively flat terrain and high values represent rugged areas. Positive TPI values represent ridges, negative TPI values represent valleys, and flat terrain or areas of constant slope are represented by a TPI value near zero. Resistance to ruffed lemur movement was greatest in flat (low value) and rugged (high value) terrain as show in panel (A). Resistance to ruffed lemur movement was greatest in areas with canopy cover under 5m as show in panel (B). Resistance to ruffed lemur movement was lowest for shallow slopes leading to ridges as show in panel (C).

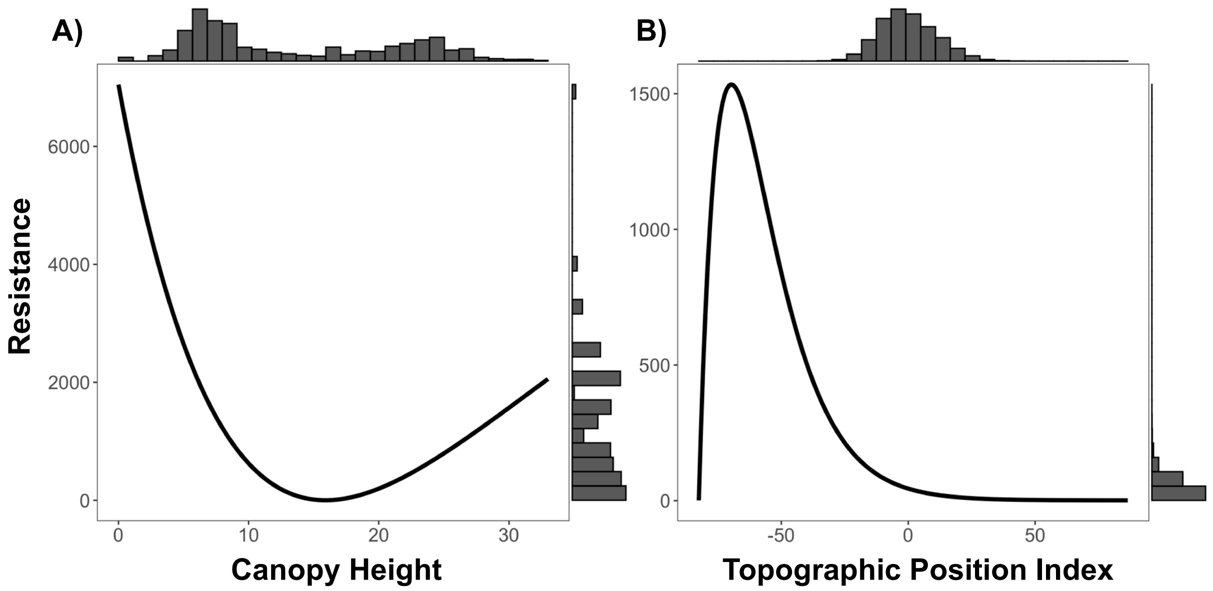

Figure S5. Resistance transformation plot for 'Composite B' surface of (A) Terrain Ruggedness Index (TRI) and (B) canopy height. Positive TPI values represent ridges, negative TPI values represent valleys, and flat terrain or areas of constant slope are represented by a TPI value near zero. Resistance for ruffed lemur movement was greatest in areas with canopy cover under 5m as show in panel (A). Resistance for ruffed lemur movement was lowest for slopes leading to ridges and ridges as show in panel (B).

Table S4. Percent contribution of each for each singular surface comprising the optimized composite surfaces.

Combination	Canopy Height	TPI†	TRI‡
Combination A	74.45	0.28	25.27
Combination B	87.87	12.13	-
Combination C	1.55	-	98.45
Combination D	-	2.98	97.02

†TPI: Topographic Position Index

‡TRI: Terrain Ruggedness Index

Table S5. Mean height, basal area (m^2/ha), stem density (stems/ha), effective number of species (ENS; exp(H')), mean IVI of *Varecia* food species, mean NDVI, and standard deviation of altitude at 15 sites in the Ranomafana region.

Site	Average Canopy Height	Basal Area	Stem Density	ENS†	Average IVI‡	Average NDVI§	Topographic Variation
AMBO	15.94	49.2	664	55.64	5.84	0.705	67.48
HG	13.23	31.6	768	45.47	6.49	0.662	63.92
MAD	10.55	34.7	756	39.51	3.97	0.742	33.12
MALA	10.68	39.2	640	48.22	2.71	0.752	117.95
MAND	11.48	31.2	604	39.42	4.53	0.754	65.27
MGV	15.19	51.6	676	83.42	4.99	0.757	124.89
SAHA	11.60	30.1	604	38.34	5.48	0.752	127.13
SAKA	12.73	16.4	360	45.13	7.90	0.737	62.00
TALA	12.65	13.8	416	45.37	8.55	0.744	47.67
TAND	13.58	52.3	688	63.31	5.45	0.746	62.23
TATA	11.42	31.9	668	55.07	5.13	0.731	130.25
VALO	12.26	30.8	564	51.91	5.30	0.749	85.43
VANA	10.12	29.1	924	41.53	6.69	0.748	96.27
VATO	12.68	38.1	720	47.13	5.53	0.742	36.26
VIAVY	12.39	27.4	724	53.95	4.74	0.735	59.11

†Effective Number of Species

‡IVI: Importance Value Index

§NDVI: Normalized Difference Vegetation Index

Table S6. Landsat scene information from composite scenes used in generating thematic maps.

Year	Collection Date	Sensor	WRS§ Path	WRS Row	Data Type	Percent Cloud Cover Land	Solar Azimuth	Solar Elevation	Resolution
1990	1989-09-20	TM†	158	075	L1TP††	0.00	62.648	47.226	30 m
	1989-09-20	TM	158	076	L1TP	0.00	61.487	46.236	30 m
	1990-11-01	TM	159	075	L1TP	1.00	84.245	54.821	30 m
1998	1997-10-28	TM	158	075	L1TP	23.00	80.179	57.696	30 m
	1997-10-28	TM	158	076	L1GS‡‡	60.00	78.125	57.203	30 m
	1998-10-06	TM	159	075	L1TP	0.00	66.926	54.398	30 m
2009	2008-10-01	TM	159	075	L1TP	0.00	63.469	54.400	30 m
	2009-11-14	TM	158	075	L1TP	9.00	87.993	62.836	30 m
	2009-11-14	TM	158	076	L1TP	5.00	85.307	62.528	30 m
2016	2015-11-15	OSI TIRS‡	158	075	L1TP	3.96	87.178	65.139	30 m
	2015-11-15	OSI TIRS	158	076	L1TP	45.95	84.227	64.808	30 m
	2016-11-08	OSI TIRS	159	075	L1TP	0.05	83.134	64.952	30 m

†TM: Thematic Mapper
‡OSI TIRS: Operational Land Imager and Thermal Infrared Sensor
§WRS: Worldwide Reference System
††L1TP: Level-1 Terrain Precision Correction
‡‡L1GS: Level-1 Geometric Systematic Correction

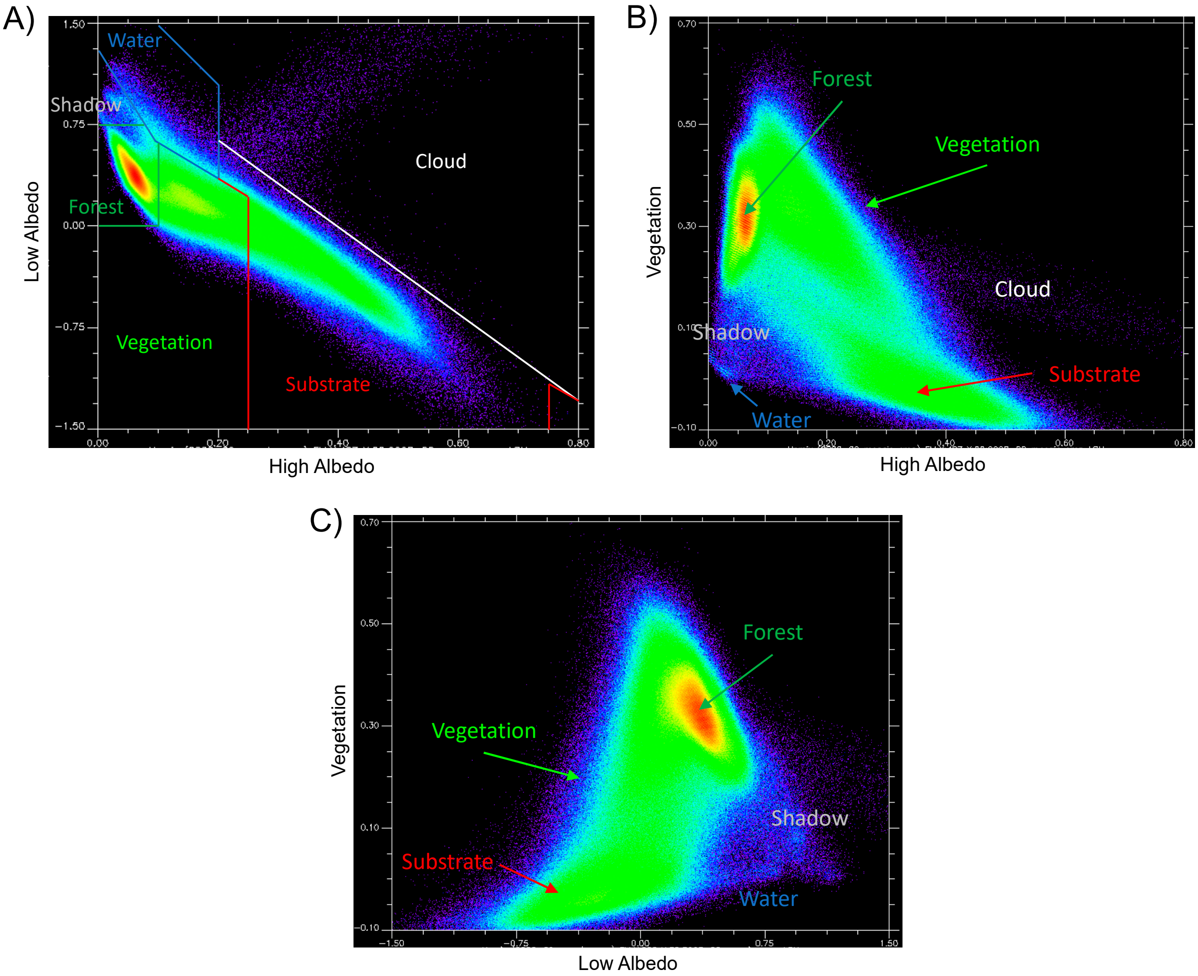

Figure S6. Two-dimensional visualizations of unmixed feature space for High Albedo, Low Albedo, and Vegetation end members from 2009 mosaic. A) High versus low albedo end members and decision boundaries for six land cover categories: forest, vegetation, substrate, shadow, water, and cloud; B) High albedo versus vegetation end members with clusters from land cover categories identified; and C) Low albedo versus vegetation end members with clusters from land cover categories identified.

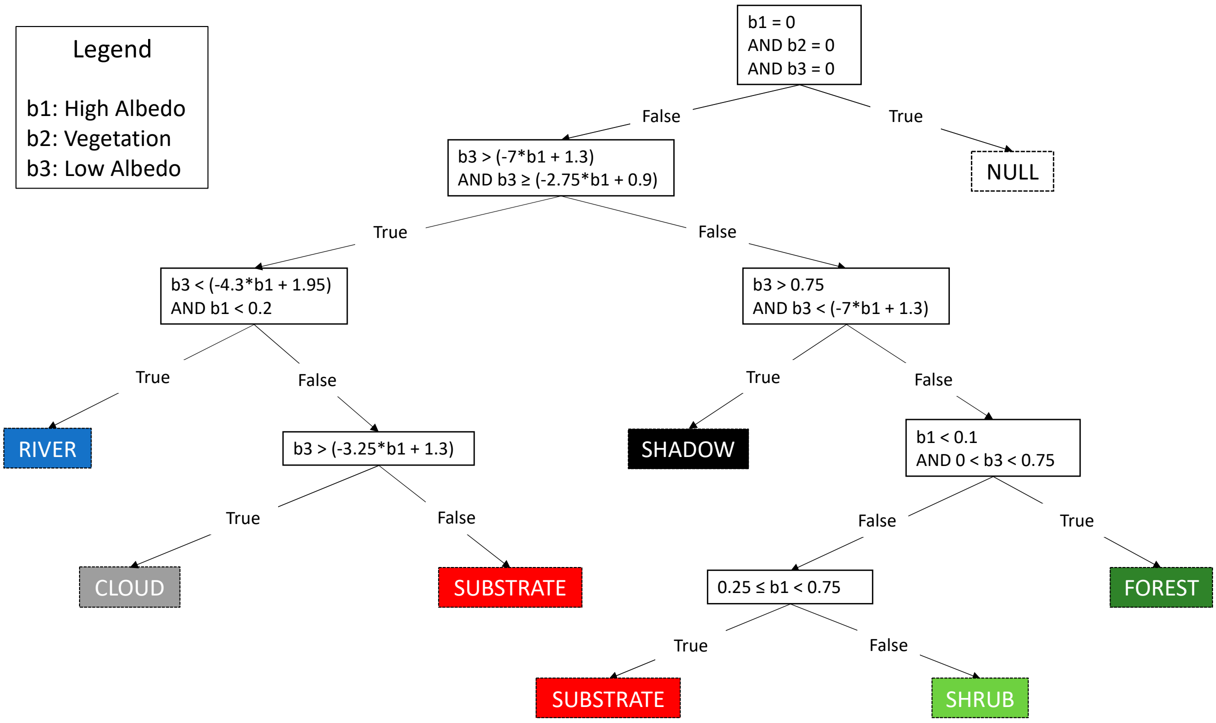

Figure S7. Customized decision tree for thematic classifications. Bands used for classification included b1: high albedo end member; b2: vegetation end member; and b3: low albedo end member.
